# Supplementary material for: The appendicular myology of Stegoceras validum (Ornithischia: Pachycephalosauridae) and implications for the head-butting hypothesis
Source: PLoS One. 2022 Sep 1;17(9):e0268144. doi: 10.1371/journal.pone.0268144 (PMC9436104; doi:10.1371/journal.pone.0268144)
Supplement: Attachment — Submitted filename: The appendicular myology of Stegoceras validum Rowe comments.docx [file pone.0268144.s005.docx]

The appendicular myology of Stegoceras validum (Ornithischia: Pachycephalosauridae) and implications for the head-butting hypothesis

Line 42: Which associated bones specifically?

Line 118: Which version of Inkscape specifically?

Line 119: Which version of Blender specifically? Versions of the software vary quite a bit.

Line 296: Shouldn’t Fig 11 be changed to Fig 3, as to reflect text order?

Line 352: Brusatte et al. 2010 needs to be listed in the references.

Line 477: Source needs to be in parentheses.

Line 478: Source needs to be in parentheses.

Line 560: Brown, 1908 must be in parentheses.

Line 755: Curry, Roger, and Forster, 2001 must be in parentheses.

Line 756: Osborn, 1905 must be in parentheses.

Line 1355: Change ‘theory’ to hypothesis.

Line 1681: There are four different Sereno citations in the text but three listed in the reference list.

All results (lines 134 – 1244) are well written; adequate sources are provided for all muscle insertions with reference to the EPB and the conservative estimates for non-avian dinosaurs are clearly stated. Though I am not completely familiar with all the literature cited (my research chiefly concerns the cranial elements of vertebrates), each piece seems to be supportive of the authors’ methods and the science is sound.

It is a nice bonus to see the carnivory in pachycephalosaurs being addressed, as well as references to extant animals, including the pelvic stresses in police dogs. These are the moments in the manuscript that particularly shine to me.
